# Anorexic Readiness Syndrome in Elite Female Acrobatic Gymnasts—International Study

**DOI:** 10.3390/ijerph192013181

**Published:** 2022-10-13

**Authors:** Ewa Polak, Adrianna Gardzińska, Maria Zadarko-Domaradzka

**Affiliations:** 1 Academic Sports Centre, Rzeszow University of Technology, 35-959 Rzeszow, Poland; 2 Institute of Physical Culture Sciences, Medical College, Rzeszow University, 35-959 Rzeszow, Poland

**Keywords:** adolescence, anorexic tendencies, cognitive competence, eating behavior, gymnastics environment, psychological education, sport participation

## Abstract

Anorexic Readiness Syndrome (ARS) is a concept used in research for the early detection of disordered eating (DE). It is a set of indicators located primarily within the cognitive and behavioral sphere of an individual’s functioning. The aim of this study was to examine whether among the elite acrobats there are girls showing a high level of anorexic tendency, and if so, what behaviors and attitudes are the most common. In addition, an attempt to determine what sport-related factors or other non-sport variables may increase the risk of ARS was conducted. The study group was made up of 133 acrobatic gymnasts aged 10–19, representing six countries that participated in the Acro World Cup competition held in Poland. The study procedures included surveys (personal questionnaire and the Eating Attitudes Questionnaire), anthropometric measurements such as weight, height, waist circumference (WC) and determination of the Body Mass Index (BMI), fat percentage (Fat%), and waist to height ratio (WHtR). A high level of ARS was found in 9.8% of acrobats. This group most often declared attitudes and behaviors indicative of anorexic tendencies. A strong relationship with the level of ARS was noted in the following: the use of fasting and diets (*p* ≤ 0.001; V = 0.54), limiting of fats and carbohydrates (*p* ≤ 0.001; V = 0.60), feeling angry after eating too much (*p* ≤ 0.001; V = 0.55), knowing the caloric value of many food products (*p* ≤ 0.001; V = 0.59), and the desire to improve the appearance of one’s body (*p* ≤ 0.001; V = 0.52). The role played in the acrobatic partnership and the region of residence were considered as the sport-related risk factors. Among non-sport factors, the strongest predictor of ARS was the age of gymnasts (β = 0.516; *p* ≤ 0.001).

## 1. Introduction

Physical activity is a prerequisite for physical health and well-being and enables the fulfilment of biopsychosocial needs at all ages. It causes a series of biological reactions in both muscles and organs, which in turn modify and regulate the structure and functions of the brain [1]. Among the physical activities recommended for children and adolescents, a special place is given to gymnastic sports, such as women’s artistic gymnastics (WAG), men’s artistic gymnastics (MAG), rhythmic gymnastics (RG), trampoline gymnastics (TG), and acrobatic gymnastics (AG). These are the sports of early specialization (attractive especially for girls) in which children undergo comprehensive and intense sports training [2,3]. Scientific studies have shown that the skills and competences acquired during gymnastic training and competitions have a positive effect on the physical [4,5,6,7,8,9,10], mental [11], social [1], and cognitive [12,13] development of a child.

Being a gymnast may also be associated with health risks [14]. One of the most serious hazards is the risk of disordered eating (DE) that may lead to full-blown eating disorders (EDs), such as anorexia nervosa or bulimia [15,16,17,18,19,20]. This type of disorder can appear at any age, but usually begins in adolescence, with anorexia nervosa at its peak in early and middle adolescence and bulimia in late adolescence [21]. Scientific studies have shown that the estimated prevalence of DE and clinical EDs in athletes is higher than in non-athletic populations and generally ranges from 0% to 19% in males and from 6% to 45% in females [22,23]. Clinical EDs are more prevalent in women compared to male athletes [24], especially in elite adolescent athletes compared to their peers in the general population [14,19,25] and in sports that depend on thinness and weight compared to other sports [25,26,27].

Elite athletes are more likely to meet the criteria of DE, which usually include subclinical conditions defined as abnormal eating habits and based on the assumption that they are in a broad spectrum between healthy and pathological eating patterns [22,27,28,29]. Previous studies have shown that, compared to the general population, athletes with EDs are less prone to psychopathology and have a better prognosis for recovery [11]. They are more likely to present disorders with specific characteristics, such as anorexia athletica [25] or orthorexia nervosa [30], than full-blown mental illnesses such as anorexia nervosa or bulimia [19,23]. Following Bachner-Melman et al. in the early 1980s, a hypothesis was made that there was an analogy between certain groups of athletes and patients diagnosed with anorexia nervosa, based on having certain common features. It was assumed that both groups are characterized by perfectionism, high self-expectations, competitiveness, repetitive exercise, compulsiveness, distortions of the body image, and preoccupation with weight and diet [11]. The results of the studies conducted since then have confirmed that sport, especially professional sport, can contribute to the occurrence of EDs, because it combines not only behavioral patterns, such as strict diet and excessive physical activity, but also personality traits such as perfectionism, competitiveness, fear of performances, and intense preoccupation with body image. The competitive nature of sport and a strong desire for excellence can lead athletes to adopt the traits of people with EDs [21]. However, the results of these studies also show that any ED is a personal response to the individual’s living conditions. The multiple interaction of biological-genetic, family, psychological, and other (e.g., sports-related) factors determines whether a person will eventually develop malnutrition and eating behavior aberrations. Furthermore, the type of sport practiced can have an influence on athletes’ unhealthy eating behaviors [24].

Teenage girls who practice aesthetic sports, especially elite gymnasts are the population particularly at risk of developing EDs. Their sense of physical attractiveness is strongly related to the mental condition and well-being [20,23,25,31,32,33]. Scientific evidence shows that practicing gymnastic sports may be a reason for greater susceptibility to risky eating behavior. In gymnastic sports, the emphasis is on thinness, weight control, and sometimes even on weight reduction of athletes, due to performance during competition depending on the subjective opinion of the judges and on aesthetic evaluation, not on measurable results [8,11,23,34]. The ability to maintain a specific body shape and weight is usually associated with a favorable aesthetic impression and optimization of the exercises performed. However, it should be remembered that too low body weight and too little adipose tissue may have a negative impact on the sports performance [33,35], as well as on health, causing injuries [36].

Gymnasts are often short and lean, and world-class gymnasts are even moderately thin. Additionally, puberty is often reported later in female gymnasts than in the general population [37]. This is because gymnasts begin intense training and specialization when they are few years old and reach their peak performance during adolescence. In this difficult developmental period, they must deal with changes related to physical growth, sexual maturation, and the demands of the sports environment [21]. The gymnastic environment made up of athletes, their families, coaches, judges, and fans place particular emphasis on thinness and controlling body image [16]. This is evidenced by external elements of the environment, which include strenuous training and performing increasingly difficult exercises, the need to put on sports clothes that reveal the body, frequent weight control, mirrors in changing rooms and training places, shared changing rooms encouraging comparison of body shape and size, discussions on methods how to lose weight, or the negative comments about weight heard from coaches, peers, and rivals. They are also often subjected to a socio-cultural pressure to be slim and a pressure from coaches to lose weight [11,16,35]. Long-term functioning in such an environment may cause cognitive disorders in gymnasts consisting in an inadequate perception of their own body with a tendency to overestimate its weight, dimensions, and proportions [16,38].

The desire to conform to the Ideal physical standards of the sport practiced means that teenage gymnasts often use low-energy diets and inappropriate nutritional strategies when trying to regulate body weight and/or body shape [20,21]. Such actions may result in micronutrient deficiencies, mainly in calcium, iron, folic acid, vitamin D, and zinc [19]. It was noticed that the results of using unhealthy methods of weight reduction in gymnasts include decreased height, weight, body fat, delayed first menstruation, and delayed bone growth. This, in turn, poses a high risk of developing relative energy deficiency (RED-S) and the female athlete triad [19,39]. It is especially harmful in adolescence, as at this age maximum bone density should be achieved and the reproductive system should develop and mature. Imbalances in bone remodeling and low body fat slow down these natural developmental processes [19]. Deficiency or lack of adequate nutrients and electrolytes also results in hormonal imbalance and the shrinkage of white and gray matter in the brain. Therefore, there are problems with cognitive functions in relation to body image, working memory, spatial memory, and cognitive flexibility [40]. A high risk of developing an ED also increases the likelihood of developing psychological problems, including low self-esteem, anxiety, and depression. Athletes with an ED are also 5% more likely to attempt suicide than those without an ED [2].

Research on DE and EDs in gymnasts provides a lot of valuable information about the specificity of this phenomenon. However, they do not allow the comparison of research results due to significant differences in methodological issues, such as different sports, levels of competition, screening tools, researchers (coaches, scientists), training periods (during and off-season), and age groups. Moreover, the undertaken research focused on various issues. Some studies analyzed the diet used by athletes [17,37,41], others were focused on identifying behaviors that indicate DE [28,32] or on psychological and cognitive risk factors [18,20,34,42].

Anorexic Readiness Syndrome (ARS) is a concept used in research on the early detection of DE in physically active populations. This concept was introduced by a Polish psychologist, Ziółkowska [38,43], who describes it as a set of symptoms suggesting abnormalities in the realization of the nutritional need and in the attitude towards one’s own body, conditioned psychologically, socially, and culturally. The creation of this construct was primarily of a preventive nature, serving as an early recognition tool of anorexic tendencies in children and adolescents [44,45]. ARS has been defined as a set of indicators located primarily in the cognitive and behavioral sphere of an individuals’ functioning and confirming the occurrence of unhealthy eating behaviors and a rigorous perception of their own body and self [45]. It is not the same as any of the mental illnesses included in ICD-11 [46] and DSM-5 [47] in the EDs category, nor should it be considered a ‘subtype’ of anorexia nervosa. It is a separate, independent unit which is the premorbid stage and the risk syndrome for full-blown anorexia nervosa, characterized by a specific set of symptoms and conditions.

The symptoms of ARS, which are manifested in the form of the so-called anorexic behaviors that are diagnostic indicators, include the following: interest in foods, their caloric value and/or counting the calories of meals consumed; excessive care for the appearance and focus on ones’ own body and comparing oneself with the ideals of beauty promoted, among others, by the media; tendency to control one’s weight and body dimensions, which is accompanied by emotional tension and a tendency to overestimate the size of one’s body; tendency to compete and perfectionism; the need for self-control; and emotional dysregulation, determined by the attitude towards food and the body [38,43].

According to Ziółkowska, the functioning of people with ARS is not unequivocally pathological—there is no life-threatening emaciation, obsessive striving to lose weight, or paralyzing fear of gaining weight. However, the behavior classified as ARS exceeds the generally accepted norm. A higher level of ARS does not determine the occurrence of anorexia nervosa but may indicate a tendency to anorexic behavior. These, in turn, may occur periodically and in a milder form than anorexia, but nevertheless may lead to the development of full-blown anorexia [43,45].

Previous studies on ARS have been carried out in groups of adult women [48] and teenage girls [49] from the general population in Poland. Research was also conducted among dancers aged 12–47 [50], ballet dancers and gymnasts aged 10–12 [33], and physically active teenage girls aged 10–17 [44] and aged 16–21 [45]. The Eating Attitudes Questionnaire (SGA-20) created by Ziółkowska [38] was used, among others, in studies carried out in a group of Polish dancers aged 11–25 [51] and a group of girls aged 15–17, including 71 girls from sports schools [52]. None of the studies to date have considered the prevalence of ARS in AG athletes.

Therefore, the aim of this study was to find out if among the elite acrobatic gymnasts there are girls showing a high level of anorexic tendencies, and if so, what behaviors and attitudes are most common in them. Additionally, an attempt was made to determine what sport-related factors or other non-sport variables may increase the risk of ARS.

## 2. Materials and Methods

### 2.1. Participants and Study Procedures

The study was conducted during the World Cup in AG (Acro World Cup), held in 2017 in Poland. The group of respondents consisted of girls representing all age categories and acrobatic competitions in which, according to the regulations in force of the Fédération Internationale de Gymnastique—FIG, women compete. These are the categories of women’s pairs, mixed pairs, and women’s groups [53].

The study procedures consisted of the following two parts: (1) filling in the personal questionnaire and the Eating Attitudes Questionnaire (SGA-20) [43] and (2) anthropometric measurements. The questionnaires were prepared in English, Polish, and Russian, and they were completed in the presence of members of the research team.

All gymnasts and their coaches were informed about the purpose and content of the study and gave their voluntary consent in writing to participate in this study. They were also informed of the possibility of withdrawing from the research at any time without giving any reason. The research was carried out in accordance with the principles of the Helsinki Declaration and with the consent of the Bioethics Committee at the University of Rzeszów (No. 9/06/2016).

The inclusion criteria in the study were: Acro World Cup participant status, female gender, consent of the coach, and voluntary consent of the respondent. A total of 159 girls were recruited to participate in the survey. As six respondents withdrew their consent to participate in the second part of the study, 154 girls participated in the anthropometric measurements. Due to their age, 21 girls who were under 10 and over 19 were excluded. Finally, for the analyses, the results obtained from 133 subjects were used. The female gymnasts were aged 10 to 19 years (mean 13.99 ± 2.29 years), with sports experience from 2 to 15 years (mean 6.17 ± 2.82 years). They represented the 6 following countries: Belarus (n(N = 9), Georgia (N = 12), Kazakhstan (N = 6), Poland (N = 70), the USA (N = 19), and Wales (N = 17). All subjects trained for a minimum of 2 h a day, 5 to 6 days a week, and presented the international level of AG. The group of 133 gymnasts included 64 ‘tops’, 23 ‘middles’, and 43 ‘bases’.

### 2.2. Surveys—The Personal Questionnaire and the Eating Attitudes Questionnaire (SGA-20)

The first part of the study consisted of completing two questionnaires with the help of members of the research team who were clarifying doubts and answering any questions. The questionnaires were translated from Polish into English and Russian by certified translators. The correct understanding of the content of the questionnaires was verified among the coaches of national teams participating in this study.

The personal questionnaire was used to collect data, such as country of residence, age, AG experience, weekly number of training sessions, an event category, and role played in the acrobatic partnership. Based on these data, three sport-related factors were determined. To assess the dependence of the values of ARS measures on the specificity of sports preparation, expressed as the event category, the subjects were divided into two subgroups. The subgroup named Groups (N = 75) included gymnasts competing in the women’s group, and subgroup named Pairs (N = 58) included those who competed in women’s pair and mixed pair. Considering the different role in the acrobatic partnership other two subgroups were designated. The subgroup Bases (N = 69) included girls from all event categories, who played the role of bases in pairs and groups categories, and middle gymnasts from women’s group category. The subgroup Tops (N = 64) included gymnasts, who played the role of top in all event categories. As the data on the country of residence showed large disproportions in the size of the groups (from 6 girls from Kazakhstan to 70 from Poland), the geographical region of residence, not the country, was chosen as the factor determining the sports environment. Gymnasts from the USA were assigned to the North America subgroup (N = 19), from Wales to the British Isles subgroup (N = 17), form Poland to the Central Europe subgroup (N = 70), and from Belarus, Georgia, and Kazakhstan to the Eastern Europe subgroup (N = 27).

The Eating Attitudes Questionnaire (SGA-20) was the tool used to determine the frequency of occurrence and the degree of intensity of anorexic tendencies. This questionnaire is a standardized tool for testing the prevalence (or absence) of ARS [43]. It consists of 20 statements relating to four variables characteristic of anorectic tendencies. These are the variables that constitute detailed measures of ARS: losing weight methods (AR_W_), attitude toward eating (AR_E_), style of parenting in family (AR_P_) and perception of own attractiveness (AR_A_). The task of the gymnasts was to mark the answer ‘yes’ or ‘no’, depending on how they responded to a specific sentence. An affirmative answer gives 1 point, and a negative one gives 0 points. The exceptions were sentences 13, 14, and 16, in which the answer ‘no’ was considered as the affirmative. For each examined person, numerical values of four detailed measures were determined by summing up points with the following specified key: ARS_W_—sentences number 1, 4, 12, 17, and 20, ARS_E_—sentences number 10, 11, and 18, ARS_P_—sentences number 5, 6, and 14, and ARS_A_—sentences number 2, 3, 7, 8, 9, 13, 15, 16, and 19. The value of the total score (ARS_TS_) was obtained by summing up the values of all detailed measures. The maximum total score that could be obtained was 20 points. A higher total score meant a greater intensity of irregularities in each sphere [43]. This questionnaire does not have statistical norms, hence the determination of the ranges for the low, medium, and high ARS level was based on the mean value and the standard deviation calculated for the entire group of respondents. It was assumed that a low ARS level was the one in which the ARS_TS_ scores were lower than the mean value of the points minus the value of the standard deviation. The mean ARS level is the one where the results are higher than the mean minus the value of the standard deviation, and lower than the sum of the mean and the standard deviation. A high level of ARS was assumed to be higher than the sum of the mean and the standard deviation.

### 2.3. Anthropometric Measurements

In the second part of this study, the anthropometric measurements were made. Height, weight, and waist circumference (WC) were assessed following the criteria of the International Society for the Advancement of Kinanthropometry [54]. The subjects were barefoot and wearing the gymnastic clothes/suits for the evaluation. Height was measured in a standing position with the head in the Frankfurt position, to the nearest 0.1 cm with a stadiometer Tanita HR-001. Weight was measured with an accuracy of 0.05 kg using foot-to-foot body composition analyzer Tanita TBF-300 (Tanita Corporation, Tokyo, Japan). Measurements made with the bioelectrical impedance method using GMON software (Medizin & Service, Chemnitz, Germany) which provided, among others, information on body mass index (BMI) values calculated as weight (kg)/height square (m^2^) and total body fat percentage (Fat%). Waist circumference (WC) was measured with a Seca 201 Type non-elastic tape (range from 0 to 205 cm; accuracy of 1 mm), as the smallest circumference between the lower edge of the costal arch and the upper crest of the ilium at the waistline level. For each of the gymnasts, the waist-to-height ratio (WHtR) was additionally calculated. It was calculated as WC (cm)/height (cm).

### 2.4. Statistical Analysis

All analyses were performed in the Statistica 13.3 software (TIBCO Software Inc., Palo Alto, CA, USA). The frequency measures (quantitative measures) were presented as percentage (%) and the number (N) of the female gymnasts tested. The descriptive statistics of numeric variables used arithmetic means (M), standard deviations (SD), and minimum (Min) and maximum (Max) values.

Due to the lack of a normal distribution of the analyzed measures of ARS confirmed by the Shapiro–Wilk Test of Normality, non-parametric tests were used in the statistical analyses. The Pearson chi-square test of independence was used to determine the relationship between the number of responses given in the surveys. The effect size measure was expressed as Cramér’s V coefficient (*V*) and was calculated as:

(1) $$V=\sqrt{\frac{x^{2}}{N\left(k-1\right)}},$$

where:

*x*^2^ is the Pearson chi-square statistic from the test,
*N* is the sample size involved in the test,
*k* is the smaller number of rows or column in contingency table.

It is interpreted as a measure of the relative strength of the association between the type of response provided and the ARS level. The coefficient ranges from 0 to 1 where 0 indicates no association, and 1 indicates a perfect association between the two variables. The interpretation of this coefficient was based on the degrees of freedom, assuming that for df = 1 Cramer’s V of 0.10 means weak, 0.30—moderate, and 0.50—large effect.

The significance of differences between analyzed subgroups was assessed using the Mann–Whitney U test (for two subgroups) and the Kruskal–Wallis test (for four subgroups). If the Kruskal–Wallis test yielded statistically significant results, multiple rank mean comparisons with Bonferroni alpha correction were used. The results of these post-hoc tests allowed to indicate which pairs of subgroups differ significantly in the values of the analyzed ARS measures. The same procedure was used to assess the significance of differences between the three levels of ARS for non-sporting factors. To identify the non-sport variable that had the greatest impact on the size of the ARS_TS_, a multivariate regression analysis with backward stepwise method was used. The assumptions for the correlation and the linear regression of predictors were verified before conducting the analyses. In the interpretation of the results, differences with *p* value ≤ 0.05 were considered statistically significant.

## 3. Results

### 3.1. Classification of ARS Expression Levels

Based on the data obtained from the Eating Attitudes Questionnaire (SGA-20), four detailed measures of risky behaviors were determined. The mean values of these measures were the following: ARS_W_ 1.52 ± 1.10, ARS_E_ 1.12 ± 1.11, ARS_P_ 1.03 ± 0.65, and ARS_A_ 3.66 ± 1.71 points. By summing up the values of the detailed measures, the ARS_TS_ values for each of the gymnasts were obtained. The mean ARS_TS_ value for the entire group was 7.33 ± 3.31 points. This ARS_TS_ value (M ± SD), rounded to the nearest whole unit, was used to delineate the point ranges defining the three following different levels of ARS expression: low, medium, and high. The following score ranges were determined: low ARS level when ARSTS reach four or less points, medium level when ARSTS ranges from 5 to 11 points, and high level when ARSTS reach 12 or more points. Using such defined criterion, the subjects were divided into groups with different levels of ARS expression. There were 30 gymnasts in the group with a low ARS level, which constituted 22.6% of the respondents. Ninety girls (67.7%) were assigned to the group with a medium ARS level, and thirteen girls (9.8%) to the group with the highest ARS level.

### 3.2. Analysis of the Responses Confirming the Risky Behavior in Relation to the ARS Levels

The next step in the results analysis was to compare the number of responses confirming the risky eating behavior of gymnasts in the groups of ARS levels (Table 1). This comparison is aimed at identifying the detailed measures in which the strength of the relationship was the highest and which could have contributed to an increase in the tendency to anorexic behavior among the surveyed female gymnasts.

Gymnasts with the high level of ARS expression provided answers confirming risky behaviors and attitudes significantly more often than girls with medium and low levels of ARS in each of the detailed measures and in all statements. In five out of twenty statements a large effect of the association with the ARS level (expressed by Cramér’s V coefficient values) was noted. Two of these behaviors were included in the ARS_W_ measure. The most prevalence of the use of fasting, diets, and food restriction (*p* ≤ 0.001; V = 0.54) as well as limiting fats and carbohydrates in the daily diet (*p* ≤ 0.001; V = 0.60), was noted in girls with a high level of ARS expression. The first of the above-mentioned behaviors was confirmed by 92.3%, and the second by 100% of respondents from this group. The frequency of such behavior decreased with the level of ARS expression. The next two statements with a large effect of association belonged to the ARS_E_ measure. The feeling of anger after eating too much (*p* ≤ 0.001; V = 0.55) and knowing the caloric value of many food products (*p* ≤ 0.001; V = 0.59) were confirmed by 100% of the surveyed girls with a high ARS level. The frequency of declaring these behaviors also decreased with the level of ARS expression. The last of the behaviors with a large association effect was included in the ARS_A_ measure. The desire to improve body appearance was expressed by 92.3% of subjects with a high, 54.5% with a medium, and 3.3% with a low level of ARS (*p* ≤ 0.001; V = 0.52).

It is also worth paying attention to the significant differences with a moderate effect, noted in responses confirming the feeling of satisfaction with being the best in many areas (*p* ≤ 0.001; V = 0.44) and regular weight and body size monitoring (*p* = 0.002; V = 0.31). The frequency of declaring both attitudes was 100% in the group with a high level of ARS. The frequency of declaring these attitudes was 100% in the group with high ARS and decreased with the level of ARS expression.

### 3.3. Relationships between ARS Levels and Sport-Related Factors

**Figure 1:**
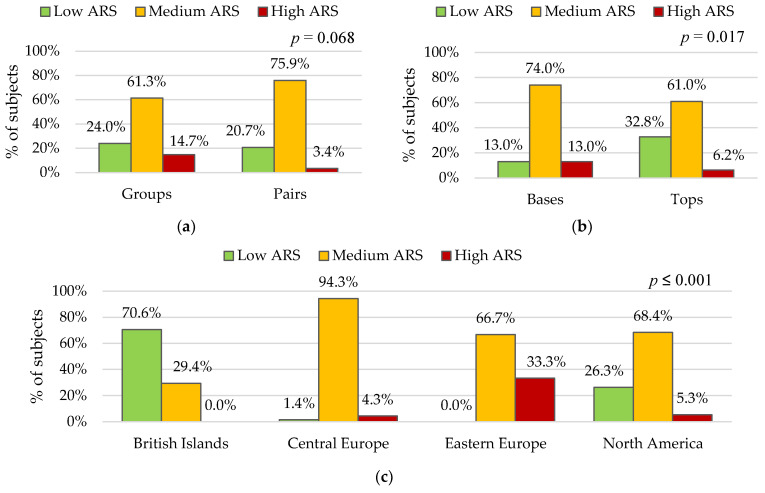
Frequency of ARS expression levels (Low, Medium, High) relative to sport-related factors: (**a**) Event category; (**b**) Role in partnership; (**c**) Region of residence. *p*—probability value calculated using the chi-square test of independence.

The next step in the results analysis involved the verification of the prevalence frequency of various levels of ARS expression against sport-related factors. The graphs in Figure 1 show the percentage distributions of the incidence of the three levels of ARS in the subgroups designated for each of the following sport-related factors: event category, role in acrobatic partnership, and region of residence.

The data presented in Figure 1a indicate that a chi-square test of independence showed that there was no significant association between event category and ARS level. The data in Figure 1b show that there are fewer gymnasts with a low and more with a medium and a high level of ARS in Bases, than in Tops. The differences in such percentage distributions are statistically significant (*p* = 0.017). The group comparison by region of residence presented in Figure 1c shows that there was significant association between region of residence and ARS level (*p* ≤ 0.001). In British Islands subgroup, low ARS expression was the most common, and a high ARS level was not achieved by any girls in this subgroup. The medium ARS level prevailed in gymnasts from other region of residence, with the highest rate noted in the Central Europe subgroup. The highest frequency of a high ARS levels was noted in the group of gymnasts from Eastern Europe. In this subgroup, none of the female athletes showed a low level of ARS expression.

To determine if sport-related factors significantly differentiated the mean values of the detailed ARS and ARS_TS_ measures, the relationships between the subgroups of three factors were assessed. The data in Table 2 presented the comparisons of ARS measures in subgroups of event category, role in partnership and region of residence.

The comparison of the mean values of the detailed measures of ARS between the gymnasts from Groups and Par shows that the type of event category significantly differentiates only the values of ARS_P_ (*p* ≤ 0.001). Low *p*-values show that the role in partnership significantly differentiated the level of ARS_P_ (*p* = 0.001), ARS_A_ (*p* = 0.001), and ARS_TS_ (*p* = 0.001), indicating higher results in the Base subgroup. The sport-related factor that significantly differentiated the values of all ARS measures was the region of residence. The values of probability calculated using post-hoc test indicate that gymnasts from the Eastern Europe compared to gymnasts from the British Islands reached higher scores of ARS_W_ (*p* = 0.002), ARS_E_ (*p* ≤ 0.001), ARS_A_ (*p* ≤ 0.001), and ARS_TS_ (*p* ≤ 0.001). They also obtained higher scores of ARS_W_ (*p* = 0.021), ARS_P_ (*p* = 0.002), and ARS_TS_ (*p* ≤ 0.001) than gymnasts from Central Europe, and higher scores of ARS_E_ (*p* = 0.019), ARS_P_ (*p* = 0.046), ARS_A_ (*p* = 0.011), and ARS_TS_ (*p* ≤ 0.001) than gymnasts from North America. The higher scores of ARS_E_ (*p* ≤ 0.001), ARS_A_ (*p* = 0.003), and ARS_TS_ (*p* ≤ 0.001) have also been in the Eastern Europe group than in the British Islands group.

### 3.4. Relationships between the ARS Level and Non-Sport Factors

In the last step of the analysis, the values of the variables characterizing the age and the physique of the subjects were compared against different levels of ARS. This analysis was aimed at verifying whether any of the above-mentioned non-sport factors could be a predictor of ARS occurrence. The data in Table 3 presented variables as M ± SD; Min–Max for each group of ARS level. The significance of the differences was assessed by the Kruskal–Wallis test, and for the variables in which it was indicated that there was a statistically significant difference, post-hoc tests were used. In this way, the variables significantly differentiated were identified.

The results presented in Table 3 show that age, weight, height, WC, and BMI significantly differentiated the levels of ARS expression of the studied gymnasts. The group with a high level of ARS was characterized by the significantly higher values of age (16.3 ± 2.6 years; *p* ≤ 0.001), weight (49.8 ± 14.4 kg; *p* = 0.013), and BMI (20.4 ± 3.26; *p* = 0.020). The significantly highest height (1.55 ± 0.12 m; *p* = 0.004) was characteristic of girls with a medium ARS level. It was also noted that a disturbingly low Fat% was present at each of the ARS levels. The lowest values of the test probability *p*-value determining the significance of differences for the age of gymnasts were noted. To verify whether athletes’ age can be a predictor to determine the risk of ARS, a multiple regression analysis was performed.

The assumptions for the correlation and the linear regression were verified before conducting the analyses. The dependent variable and all predictor variables were quantitative. The normal distribution has been confirmed based on values of skewness and kurtosis. All the predictor variables were not correlated very highly. The assumptions of multicollinearity were verified using the variance inflation factor (VIF). Only variables showing a VIF of less than 10 were included in the regression model. In the selected model the dependent (explained) variable was the ARS_TS_, and the independent variables (predictors) were age, WHtR, BMI, and Fat%. Results of the multiple linear regression indicated that there was a moderate collective significant effect between the Age, WHtR, BMI, Fat%, and ARS, (F(1, 131) = 47.5, *p* ≤ 0.001). R square (R^2^) equals 0.266, and adjusted R^2^ equals 0.260. This means that this model explains about 26% of ARS_TS_ variability in the study group. The coefficient of multiple correlation (R) equals 0.516, which means that there is a moderate correlation between the predicted and the observed data of ARS_TS_. The results of the analyses using backward stepwise method show that the independent variables WHtR, BMI, and Fat% were excluded from the regression model. They were not significant as predictors for ARS_TS_. The only one predictor significantly explaining ARS was the age of studied gymnasts. Interpreting the standardized β regression coefficient, it was found that if the age of gymnasts is one year higher, ARS_TS_ will increase by about 0.52 points (β = 0.516; *p* ≤ 0.001).

## 4. Discussion

AG is a collaborative sport practiced by both male and female athletes. Acrobatic training experience begins in early childhood to achieve a high level of performance in adolescence. According to the rules set out in the FIG regulations, during the competition, female acrobatic gymnasts compete in pairs (as a top or a base in women’s pairs and as a top in mixed pairs) or in groups (as a top, a middle, or a base in women’s group, consisting of three gymnasts). Male acrobatic gymnasts compete in pairs (as a top or a base in men’s pairs and as a base in mixed pairs) or in groups (as a top, a middle, or a base in men’s group consisting of four gymnasts). During competition they perform the three following types of exercises: balance, dynamic, and combined, each with their own characteristics. All exercises must be performed to music, and they must contain a harmonious combination of individual elements (acrobatic series on the floor) and collective elements (static pyramids, holds, grips, and throws) in perfect synchronization with choreography [53,55]. Acrobatic female and male gymnasts are characterized by specific morphological profiles depending on the role they play—top or base. The tops are younger, shorter, and lighter, and perform a variety of static elements assisted by the bottom ones in team arrangements, or large acrobatic jumps when thrown over the bases with re-landing on their body or on the floor. The bases are older, taller, and tend to be higher in weight and fat content as their role is to support and grip the tops which require more strength and endurance [55,56,57]. Top gymnasts should also be strong and must not be afraid of heights and be able to perform acrobatics with a great kinesthetic sense [58]. Each of the individual gymnast’s size and skill are used to complement each other in the partnership.

Most of the studies on nutritional patterns and attitudes in gymnasts has focused on athletes practicing WAG or MAG [8,16,27,34] and RG [20,59]. Only a few authors included in the studied groups gymnasts practicing TG or AG [11,15,17,21,37,42]. The knowledge about the risk of DE among acrobatic gymnasts is very limited so far. The first study in which this group of athletes was included was carried out by Nordin, who compared the occurrence of DE in UK athletes aged 10–15 years from three gymnastic sports WAG (N = 17), RG (N = 17), and AG (N = 16). He identified four female rhythmic and three acrobatic gymnasts as at risk of EDs [15]. In recent years, the next studies focused on the assessment of eating behavior and body image among AG athletes from Portugal [17] and Spain [18,42,60] have been published.

The results obtained in our study showed that 9.8% of the studied acrobats showed a high, and 67.7% a medium level of ARS expression. In 22.6% of the surveyed gymnasts, a low level of ARS was found, which meant no tendency to anorexic behavior. Such a percentage distribution is like the results of studies carried out among 92 Polish dancers aged 11–25, which showed that 12% of dancers had a high level and 57% had a medium level of ARS [51]. Anorexic tendencies were not displayed by 25% of the surveyed dancers. In another study conducted among adolescent girls aged 15–17 years from Polish sports schools, 12% of the respondents were girls with a high, 67.6% with a medium and 19.7% with a low ARS level [52]. Therefore, the frequency of high levels of ARS in the studied acrobatic gymnasts was lower than in Polish dancers and sports schools’ students. The occurrence of high levels of ARS expression in the elite acrobatic gymnasts that were examined in this study is also lower than the occurrence of EDs in elite female athletes of other gymnastic sports. In British [15] and Greek [8] studies, in which teenage female gymnasts from various sports were combined into one group, EDs were found in 12.3% of respondents, and in studies of rhythmic gymnasts from Greece [20], in 41.5% of elite and 14.6% of recreational gymnasts. Walter et al., who studied German elite male and female athletes from various sports, found that the highest incidence rate of clinically significant nutritional pathology (9.6%) was found in female athletes aged 15–18 years involved in high-risk sports [34]. However, it should be emphasized that the above-mentioned studies were performed with the use of other tools, such as the Eating Disorder Inventory (EDI; 10) [15], the Eating Attitudes Test (EAT-26) [8,20], and the Eating Disorder Examination Questionnaire for children [34] and in groups with a slightly different age range; 10–15, 11–17, 13–15, and 13–18.

Our study was also focused on finding the risky attitudes and behaviors of elite gymnasts with a high level of ARS. Significantly, the highest percentage of respondents from this group confirmed all behaviors included in the Eating Attitudes Questionnaire (SGA-20) as detailed measures of AR_W_ and AR_E_. Limiting fats and carbohydrates in the diet, knowing the caloric value of many food products, and feeling angry after eating too much food was declared by 100% of respondents with a high ARS level. Moreover, 92.3% of the respondents from this group declared the use of fasting, diets, and food restriction, 84.6% vigorous exercises, and 53.8% paying attention to the quantity and quality of food. Such behaviors indicate trends that are diagnostic indicators of ARS, defined as risky interest in foods and their caloric value, and emotional tension determined by the attitude to food [38]. The third detailed measure, which determines risky style of parenting in family was ARS_P_. Among statements included in ARS_P_, all subjects with a high ARS level declared feeling satisfied with being the best in various areas, and 61.5% of them confirmed that they were controlled, admonished, or criticized by their parents. This is consistent with the ARS diagnostic indicators defined as competitive and perfectionist propensity and the need for self-control. The first of these attitudes results from participation in sport. Acrobatic gymnasts are a selected group of girls for whom participation in sport is a way to fulfil the need for competition and perfectionism. Without such attitudes, it would not be possible for them to achieve sports success. In turn, feeling pressure from parents may have an impact on undertaking risky eating behavior. It all depends on the relationship of the gymnast with her parents. It is important whether the parents support gymnast in her quest for success or whether they instruct and severely criticize. The influence of the relationship with parents on a high level of ARS expression was also demonstrated in a study conducted in Polish teenagers aged 12–14. This study results that a high level of criticism from parents and their high expectations coexisted with a high level of ARS in teenage girls [49]. Another study conducted in a group of British athletes aged 16–36 years (20.95 ± 3.67) found significant associations between the quality of the parent-athlete and coach–athlete relationship and nutritional psychopathology. In particular, the relationship between the parents and the coach and the athlete, characterized by increased conflict and decreased support, was indirectly related to the severity of nutritional psychopathology, through low self-esteem, increased self-critical perfectionism, and depression [61]. There is also a risk that rigorous self-evaluation may promote unhealthy eating behavior and consequently adversely affect nutritional status and pose a risk of developing EDs that usually begin in early adolescence and may be revealed or worsen later [33].

The most chosen statements were those included in the ARS_A_ measure, which defined the respondents’ attitude to their attractiveness and their own body image. Attitudes proving that physical attractiveness was assigned high importance were most often noted among girls with a high ARS level. As many as 84.6% of respondents from this group stated that appearance is of great importance in achieving success in life, 76.9% believed that men prefer slim women, and 38.5% declared comparing themselves to models and famous actresses. The only behavior from the ARS_A_ detailed measure, confirmed by 100% of the subjects in the high ARS group, was weight and body size control. However, such behavior cannot be considered a manifestation of the ARS. The proper performance of the pair/group exercises depends on the physical preparation of both the bases and the tops, but also on the proportion of differences in weight and height between them. The increase in height or weight of one of the gymnasts changes these proportions and forces the others to change the technique of performing exercises. So, maintaining the optimal weight and body size of a top is essential for the pair/group to achieve high performance and success during the competition. Therefore, regular control of weight and body dimensions is one of the specific behaviors of elite acrobatic gymnasts and indicate a sense of responsibility for the entire pair/group. However, if it does not cause anxiety and stress in gymnasts, it should not be interpreted as a risky behavior, but as one of the ‘professional duties’ resulting from practicing this sport. This is confirmed by the results of our study, in which a spike in the percentage of respondents declaring such behavior was also noted in groups with medium and low levels of ARS. Respectively, it was 87.8% and 63.3%. Similar proportions were noted in the respondents’ declarations about the attention they pay to taking care of themselves and their appearance. This behavior was confirmed by 92.3% of gymnasts with a high, 72.2% with a medium, and 46.7% with a low level of ARS. Our results are consistent with the conclusions of previous studies which proved that elite gymnastics is characterized by a subjective and aesthetic evaluation. With increasing technical requirements, it imposes strict requirements on body shape and proportions throughout the international gymnastic community [11,15,28,29,35]. Moreover, if caring for one’s appearance aims to adapt to sporting requirements and does not take on an obsessive form, it should be interpreted as fulfilling ‘professional duties’ in sport.

According to Ziółkowska, such attitudes are typical for ARS and differ from the clinical symptoms of anorexia nervosa. In her opinion, the self-esteem of people with ARS is usually underestimated in self-attractiveness, but not generalized, which means that a young girl may feel inferior to others when it comes to appearance, but assesses herself much better in other areas, e.g., intellectual achievement, or social successes. In the case of anorexia nervosa, the situation is the opposite; the self-esteem of patients is drastically underestimated in all areas, except for weight loss, which becomes a source of self-esteem [38,43]. Our research noted that approximately 80% of all surveyed gymnasts consider themselves to be liked by others and thought of as attractive, which confirms high self-esteem regardless of the level of ARS expression. Even so, body image attitudes were not so positive. As many as 61.5% of the surveyed gymnasts with high ARS considered their body disproportionate, and 46.2% declared frequent feeling of bad mood and dissatisfaction with themselves. As many as 92.3% of girls with a high level and 54.5% with a medium level of ARS declared their willingness to improve their appearance. Similar trends were observed in a study of Spanish acrobats aged 10–18 years [18]. It was shown that all studied gymnasts declared satisfaction with the appearance of their bodies, and at the same time, 41.4% of them wanted to have a better figure. Other studies also conducted among Spanish acrobatic gymnasts found that 75.3% of 81 subjects aged 10–19 were satisfied with their body image [42]. Another Spanish study reported that 61% of acrobatic gymnasts were satisfied with their BMI, but 50% showed body image distortions. The dissatisfaction rate was 61% for female athletes and was higher than for male athletes [60]. This means that female acrobatic gymnasts are usually characterized by a high self-esteem of their own attractiveness, while being dissatisfied with their body image.

The second aspect of our study was to determine which of the analyzed factors significantly differentiate the ARS level in elite acrobatic gymnasts. Previous studies among teenage athletes have shown several risk factors for the development of EDs. For example, Walter et al. presented a dominant relationship between the type of sport practiced, social pressure, and personality factors with the symptoms of EDs. The following risk factors were being in the age range of 15–18, being a woman practicing high-risk sports, as well as the psychological connection of success by athletes to weight loss, and athletes experiencing social pressure on nutrition and body shape [34]. Brown et al. list other specific behaviors or risk factors, which include a history of critical comments about food or weight from an influencer, history of depression, history of diet, obsessive or perfectionist personality, frequent fluctuations in weight or pressure to lose weights, starting sports early, overtraining, recurring injuries, and inappropriate coaching behavior [39]. On the other hand, Kontele et al. claim that the high risk of EDs in gymnasts is caused by factors such as daily intensive training and the associated high nutritional requirements, the young age of reaching the peak of sports performance or special standards applied in the sports environment. In AG, as in other gymnastic sports, where optimal performance depends on the ability to overcome gravity and move the body rapidly through space, reduced body weight and especially fat mass are often considered a ‘benefit’. In combination with maximum muscle strength, it improves flexibility, strength, and exercise technique, and increases the power-to-weight ratio [37].

In this study, the identification of risk factors for ARS was based on sport-related and non-sport variables related to development and body composition. Sports-related risk factors include playing the role of base and practicing AG in the Eastern Europe region. It was shown that in the studied group of elite athletes, a high level of ARS was noted significantly more often in the bases (13%) than in the tops (6.2%) and the most often in the subgroup gymnasts from Eastern Europe (33.3%). The mean value of the measure, which was a determinant of the ARS expression level (ARS_TS_), significantly differentiated the role in partnership (*p* = 0.017) and the region of residence (*p* ≤ 0.001), indicating higher results in bases and Eastern European gymnasts.

The greater susceptibility to ARS among the bases can be explained by the fact that the ideal body shape in AG requires being thin and light but is dependent on the role played in acrobatic partnership [15,56]. Research to date suggests that tops are much shorter, lighter, and less fat than base acrobats, and the level of competition contributes to these differences [55]. Natural developmental changes, especially sexual development in women, can be viewed as undesirable and even detrimental to the performance of tops. It becomes problematic and difficult for both the female athlete herself and the teammates [21]. It may also be a problem in adapting to the rules of participation in competitions set out in the regulations and Code of Points established by FIG [53]. Because these regulations define the age limits of athletes who can compete in individual age categories, as well as the permissible proportions of height between the partners in a pair or in a group. If the difference in height between the partners is 30 cm or more, the pair or group points are deducted from the final score of each exercise performed during the competition [53]. Increasing height and weight, as well as changing the proportion and body shape in a top gymnast does not have to mean the end of a sports career. Elite AG requires the cooperation of gymnasts of different ages and physiques. This allows them to switch from top to base roles and pursue a sports career with another pair or group.

However, it should be remembered that acrobatic gymnasts function as interdependent units because success in AG can be achieved in pair or group only. For this reason, and due to the different combinations of gender, age, and number of gymnasts cooperating in each of the event categories, the psychological pressure on acrobats is higher than on gymnasts who train and compete alone [21]. As previously mentioned, sports psychological challenges are beneficial for building cognitive competences in adolescence. They allow teens to shape both positive responses and the ability to generate appropriate coping strategies to deal with the stresses of sport and everyday life [62]. However, unrealistic expectations from a parent or a coach, pressure from the coach, relatives, or sport partners to keep low body weight, and a lack of professional counselling can lead to lowered self-esteem, mental overload, depression, anxiety, and potential burnout, worsening athletic performance [2,62].

As the previous study showed, the functioning of teenage female athletes in a training environment with a high-pressure in relation to weight and body composition may increase the risk of DE [2]. In our study, this relationship was confirmed by proving that the region of residence significantly increases the level of ARS. Therefore, the place of residence in which the studied gymnasts train can be considered a risk factor for ARS. This factor is strongly related to the psychosocial gymnastic environment, which can include a variety of coaching styles, parental pressure, intense practice and demanding competition, social isolation, and lack of opportunities for social development, public display of skills and evaluation by others, and in some cases living and training outside the home and family. It is also not without significance that elite gymnastics has a tradition (derived from the Eastern European sports subculture) of being both paternalistic and authoritarian, with a high level of external control of athletes [31]. While gymnasts appear to have remarkable ability to cope with pressure, the pressure of their daily training environment may be overwhelming for some of them [63]. A supportive environment, that encourages athletes to do the best of themselves is safer than one where criticism and bullying (especially in relation to weight and body composition) are the norm [26].

The comparison of age differences and anthropometric measures and indices showed that the group with a high level of ARS was characterized by the significantly highest values of non-sport variables, except for height. The mean values of the age and anthropometric measures and indices of acrobats in this group were: 16.3 ± 2.6 years of age, 49.82 ± 14.38 kg of weight, 1.54 ± 0.14 m in height, 64.6 ± 7.9 cm of WC, 0.42 ± 0.03 WHtR, 20.4 ± 3.26 BMI, and 19.3 ± 8.6 Fat%. The mean values of these variables are very similar to those obtained in the study of Spanish acrobatic gymnasts by Peláez-Barrios and Vernetta. The Spanish acrobatic gymnasts were younger (13.7 ± 3.1 years), had a similar height (1.54 ± 0.11 m), less weight (46.42 ± 10.25 kg), and had smaller WC (59.8 ± 7.1 cm), WHtR (0.38 ± 0.03), and BMI (19.5 ± 3.26). The only variable in which a slightly higher mean value was noticed than in the group of elite gymnasts studied by us was Fat% (19.5 ± 4.4) [42].

The analysis of the relationship between the values of the above-mentioned variables and the values of ARS_TS_ allowed us for the identification the age of studied gymnasts as the non-sport predictor of ARS. It was shown that if the age of gymnasts is one year higher, ARS_TS_ will increase by about 0.52 points. This is in line with the results of other studies which emphasized that starting intensive training in childhood is associated with the onset of EDs later in life [11,27], and the onset of puberty is one of the main triggers of EDs [15]. Attention was also paid to the relationship between early sports specialization and negative psychological indicators, such as EDs [15,28]. This is because gymnastic sports are sports where intense training begins very early, but the peak of sports performance comes in adolescence, when children experience major physical and emotional changes [42]. Moreover, adolescence is a period of high sensitivity to body image and a time important for personality development and the establishment of eating habits [19,32,64]. Like other gymnasts, teenage acrobats also must cope with changes related to physical growth and sexual maturation as well as the demands of intensive training and the expectations of the gymnastic community [8]. However, it was emphasized that this relationship may reflect elements related to the aesthetic component of these sports, rather than being a direct consequence of specialization per se [3].

However, this study has some important limitations. Firstly, the study used the Eating Attitudes Questionnaire (SGA-20) as research tool, which is little known in international studies. This questionnaire was developed in 2000 by the Polish psychologist, Ziółkowska, who in 2018 proposed a new form of this questionnaire, called SGA-12 [44]. This modified ARS assessment tool (SGA-12) should be used in future research. Secondly, gymnasts from various countries participated in this study, but the largest group were girls from Poland. This group structure is not representative of the international elite in AG, which may be considered in future studies on the prevalence of ARS. The structure of the group in our study was determined by the composition of the national teams participating in the Acro World Cup competition, held in Poland. Thirdly, due to the tight schedule of the competition, some gymnasts did not agree to participate in the study, what resulted in disproportions in the size of the national groups. This forced the authors to include some of the respondents into multinational groups, defined as groups of the region of residence. Future research should be carried out in larger national groups.

To better understand the range of occurrence and specificity of ARS, future research should consider the influence of the AG environment, including coaches, parents and even judges in the study group. They should also consider more psychological and cognitive aspects, as the etiology of ARS is multifactorial. It is also worth using other standardized tools to study EDs in AG athletes.

## 5. Conclusions

Early diagnosis and implementation of preventive actions related to DE in young athletes is an important aspect of their physical and mental health. This study provided the first information about the ARS phenomenon in the international environment of elite AG. The obtained results allowed for the identification of attitudes and behaviors that are early symptoms of developing anorectic tendencies in 9.8% of teenage acrobatic gymnasts. We also found that sport-related factors and age may contribute to the intensification of this tendences.

These results can be an indication of what competences should be developed and what educational activities should be undertaken to prevent disturbed eating behavior among adolescents practicing acrobatic gymnastics. Providing appropriate prophylaxis through education in the field of proper eating behavior and weight loss techniques, and the emphasis on body acceptance, will allow acrobatic gymnasts to maintain healthy body functions, reduce the incidence of Eds, and be in the best shape for their sport. Activities undertaken in this sport should also aim at improving the awareness of parents, coaches, and judges regarding the impact of low-weight pressure on the physical and mental health of young acrobatic gymnasts.

## Figures and Tables

**Table 1:** Percentage distribution of responses confirming risky behavior in the detailed measures of the Eating Attitudes Questionnaire in relation to ARS levels.

Behaviours and Attitudes Determining Anorexic Tendencies (No. in the SGA-20)	Low ARS % (N)	Medium ARS % (N)	High ARS % (N)	*p*	V
**Losing weight methods—ARS_W_**					
Use of fasting, diets, food restriction (1)	0.0 (0)	26.7 (24)	92.3 (12)	≤0.001	0.54
Taking laxatives (4)	0.0 (0)	3.3 (3)	7.7 (1)	0.379	0.12
Taking weight loss supplements/appetite suppressants (12)	0.0 (0)	0.0 (0)	15.4 (2)	≤0.001	0.41
Practicing intense physical exercise (17)	33.3 (10)	63.3 (57)	84.6 (11)	0.002	0.30
Limiting fats and carbohydrates (20)	10.0 (3)	73.3 (66)	100.0 (13)	≤0.001	0.60
**Attitudes toward eating—ARS_E_**					
Paying attention to the quantity and quality of food in the household (10)	3.3 (1)	40.0 (36)	53.8 (7)	≤0.001	0.35
Feeling angry after eating too much (11)	0.0 (0)	43.3 (39)	100.0 (13)	≤0.001	0.55
Knowing the caloric value of many foods (18)	3.3 (1)	43.3 (39)	100.0 (13)	≤0.001	0.59
**Style of parenting in family—** **ARS_P_**					
Feeling of being criticized/admonished/controlled by parents (5)	0.0 (0)	22.2 (20)	61.5 (8)	≤0.001	0.39
Feeling satisfied with being the best in many areas (6)	46.7 (14)	86.7 (78)	100.0 (13)	≤0.001	0.44
Not feeling fully accepted by parents (14)	0.0 (0)	2.2 (2)	15.4 (2)	0.019	0.24
**Perception of own attractiveness—** **ARS_A_**					
Perceiving attractive appearance as a condition for achieving success in life (2)	3.3 (1)	47.8 (43)	84.6 (11)	≤0.001	0.47
Paying considerable attention to taking care of oneself and one’s appearance (3)	46.7 (14)	72.2 (65)	92.3 (12)	0.005	0.28
Belief that thinner women are more attractive to men (7)	26.7 (8)	56.7 (51)	76.9 (10)	0.003	0.30
Frequent mood swings and feelings of dissatisfaction with oneself (8)	3.3 (1)	21.1 (19)	46.2 (6)	0.004	0.29
Comparing oneself to models and attractive actresses (9)	3.3 (1)	21.1 (19)	38.5 (5)	0.016	0.25
Seeing one’s own body as disproportionate (13)	13.3 (4)	14.4 (13)	61.5 (8)	0.001	0.36
Monitoring one’s own weight and body size (15)	63.3 (19)	87.8 (79)	100.0 (13)	0.002	0.31
Seeing oneself as unattractive to others (16)	20.0 (6)	16.7 (15)	15.4 (2)	0.899	0.04
The desire to improve the appearance of one’s own body (19)	3.3 (1)	54.4 (49)	92.3 (12)	≤0.001	0.52

ARS—Anorectic Readiness Syndrome. *p*—probability value calculated using the chi-square test of independence (2, N = 133). V—Cramér’s V for df = 1.

**Table 2:** The differences in the ARS measures in relation to three sport-related factors.

	**Event Category**				***p* ^1^**	
	Groups (N = 75)		Pairs (N = 58)			
ARS_W_	1.5 ± 1.1		1.6 ± 1.1		0.722	
ARS_E_	1.0 ± 1.1		1.3 ± 1.1		0.146	
ARS_P_	1.2 ± 0.7		0.8 ± 0.6		≤0.001	
ARS_A_	3.9 ± 1.7		3.3 ± 1.6		0.075	
ARS_TS_	7.6 ± 3.6		7.0 ± 2.9		0.480	
	**Role in Partnership**				***p* ^1^**	
	Bases (N = 69)		Tops (N = 64)			
ARS_W_	1.6 ± 1.2		1.4 ± 1.0		0.211	
ARS_E_	1.2 ± 1.1		1.0 ± 1.1		0.195	
ARS_P_	1.2 ± 0.6		0.8 ± 0.6		0.001	
ARS_A_	4.1 ± 1.6		3.2 ± 1.7		0.001	
ARS_TS_	8.2 ± 3.3		6.4 ± 3.1		0.001	
	**Region of Residence**				***p* ^2^**	Post-Hoc Test*
	British Islands (N = 17)	Central Europe (N = 70)	Eastern Europe (N = 27)	North America (N = 19)		
ARS_W_	0.9 ± 0.8	1.4 ± 1.1	2.1 ± 1.1	1.7 ± 0.9	0.001	EE–BI; EE–CE
ARS_E_	0.1 ± 0.2	1.2 ± 1.0	1.9 ± 1.1	0.8 ± 1.0	≤0.001	CE–BI; EE–BI; EE–NA
ARS_P_	0.9 ± 0.2	0.9 ± 0.6	1.6 ± 0.8	0.9 ± 0.3	≤0.001	EE–CE; EE–NA
ARS_A_	2.3 ± 0.9	3.8 ± 1.7	4.6 ± 1.4	3.1 ± 1.6	≤0.001	CE–BI; EE–BI; EE–NA
ARS_TS_	4.2 ± 0.8	7.2 ± 3.1	10.2 ± 2.9	6.4 ± 2.8	≤0.001	CE–BI; EE–BI; EE–CE; EE–NA

ARS_W_—Losing weight methods, ARS_E_—Attitude toward eating, ARS_P_—Style of parenting in family, ARS_A_—Perception of own attractiveness, ARS_TS_—ARS total score. Data are presented as M ± SD. *p*^1^—probability value calculated using the U Mann–Whitney test. *p*^2^—probability value calculated using the Kruskal–Wallis test H (3, N = 133). BI—British Islands, CE—Central Europe, EE—Eastern Europe, NA—North America. Post-hoc test*—The pairs of regions of residence with significantly different mean values.

**Table 3:** Differences in age as well as anthropometric measures and indices in relation to ARS levels.

	Low ARS (L) (N = 30)	Medium ARS (M) (N = 90)	High ARS (H) (N = 13)	*p*	*p* ^1^
Age (years)	12.5 ± 1.7; 10–17.3	14.1 ± 2.1; 10–19	16.3 ± 2.6; 11.6–18.9	≤0.001	H − M = 0.040 H − L ≤ 0.001 M − L ≤ 0.001
Weight (kg)	38.6 ± 10.0; 24.1–63.3	46.0 ± 12.9; 22.4–75.4	49.8 ± 14.4; 25.9–69.1	0.013	H − L = 0.040 M − L = 0.031
Height (m)	1.47 ± 0.10; 1.32–1.65	1.55 ± 0.12; 1.25–1.75	1.54 ± 0.14; 1.29–1.72	0.004	M − L = 0.003
WC (cm)	60.6 ± 4.9; 53–73	63.7 ± 6.3; 50–80	64.6 ± 7.9; 50–73	0.028	M − L = 0.050
WHtR	0.41 ± 0.02; 0.38–0.46	0.41 ± 0.02; 0.35–0.47	0.42 ± 0.03; 0.37–0.47	0.560	-
BMI	17.7 ± 2.3; 13.8–23.8	18.6 ± 2.8; 14.1–25.4	20.4 ± 3.3; 14.4–24.1	0.020	H − L = 0.017
Fat%	14.6 ± 6.6; 1.9–28.1	17.5 ± 8.1; 1.0–35.9	19.3 ± 8.6, 2.6–29.7	0.121	-

WC—waist circumference; WHtR—Waist to Height Ratio, BMI—Body Mass Index, Fat%—Body Fat Percentage. Data is presented as M ± SD; Min-Max. *p*—probability value calculated using the Kruskal–Wallis test: H (2, N = 133). *p*^1^—probability value using the post-hoc test for pairs of ARS levels with significantly different values.

## Data Availability

The data of the present experimental study can be available from the corresponding author via the reasonable request.
